# Ku circles the telomere?

**DOI:** 10.18632/aging.100321

**Published:** 2011-04-26

**Authors:** Christian Sell

**Affiliations:** Department of Pathology, Drexel University College of Medicine, Philadelphia, PA

Not all tumor cells are created equal when it comes to telomere biology. Tumor cells that do not express telomerase appear to utilize a telomerase-independent process to maintain telomere length, known as ALT for alternative lengthening of telomeres [1]. Although the existence of this process has been recognized for some time, the precise mechanism by which telomeres are maintained in these cells has been difficult to determine [2]. A recent report introduces a new element into the ALT story, implicating the Ku70/80 protein heterodimer (simply referred to as Ku) in the ALT process. Work reported by Li and coworkers in this issue of Aging, reveals a novel requirement for Ku in maintaining telomeres in immortal cells that utilize ALT [3]. The authors present data using a gene targeting approach to deplete both Ku subunits in two independent ALT cell lines. The ALT cells succumb to a combination of senescence and apoptosis without loss of telomere length or single-stranded telomere overhang. Surprisingly, the production of extra chromosomal DNA circles (t-circles) is reduced following Ku depletion as it is following depletion of MRE11/NBS1, known requirements for t-circle formation [4]. The results are striking because the Ku heterodimer is a central element in the nonhomologous end joining (NHEJ) DNA repair pathway, as it binds preferentially to free DNA ends and functions to recruit components of NHEJ DNA repair such as DNA-dependent protein kinase (DNAPK) and ligase IV. Although the Ku heterodimer is intimately involved in DNA repair, it has become apparent that Ku also participates in a wide variety of functions related to genome integrity. For example, Ku has been localized to origins of replication, and has been implicated in chromatin remodeling required for transcriptional activation and in telomere maintenance [5]. Ku also appears to play a role in aging. Deletion of the Ku 80 gene leads to an immune-deficient phenotype due to loss of proper VDJ recombination, but also induces a premature aging phenotype [6]. Ku 80 levels and DNA end binding also show a striking exponential correlation with species lifespan [7], suggesting that increased Ku function is requisite for long-lived species. Additionally, Ku levels decrease during replicative senescence [8]. Consistent with a higher requirement for Ku function in long-lived species, Ku appears to play an essential role in human cells while it is dispensable in rodent cells [9]. Ku has also been identified as a nodal point in systems analysis of aging-related DNA repair genes [10]. The Ku heterodimer is required for proper telomere function in multiple species, but the precise requirement for Ku seems to depend upon the specific telomere biology of the species [11]. Nonetheless, Ku appears to be an essential element of the protein complex that forms at the telomere. Ku is required for proper telomere maintenance in normal human cells and in telomerase positive cells [9, 12]. Interestingly, the role for Ku differs in each of these settings. In normal human fibroblasts, a reduction in Ku induces a rapid senescence combined with a decreased binding of a key telomere binding protein, TRF2, to the chromatin. In telomerase positive tumor cells, apoptosis is induced. Most surprising is the contrasting effect of Ku targeting on the t-circles that are diagnostic of the ALT mechanism [13]. Depletion of Ku in telomerase positive cells leads to the production of t-circles while the work of Li et al. demonstrates that depletion of Ku in ALT cells leads to a reduction in t-circles. In normal human fibroblasts Ku appears to be critical to proper cell cycle progression as cells rapidly senesce following Ku depletion. This rapid senescence likely precludes the development of either the t-circle formation or telomere fusions seen in the immortal cells. A different scenario occurs in ALT cells. In these cells, it appears that Ku has been incorporated into the mechanism responsible for t-circle production, leading to their reduction following Ku depletion. What is the common denominator between these cell types linking Ku function to telomere function? One possibility is the association between Ku and core telomere-associated proteins such as TRF2. Ku 70 has been found to directly interact with TRF2 [14]. TRF2 appears to function as a hub for the formation of specific protein complexes at the telomere [15] and the interaction between TRF2 and Ku may be important to prevent NHEJ at the telomere [16]. Depletion of Ku leads to reduced TRF2 binding to chromatin [12], suggesting that Ku may stabilize TRF2-mediated protein complexes. Given that altering TRF2 function influences t-circle formation [17], it may also be that Ku influences TRF2 protein complexes at the telomere that are necessary for t-circle formation. In addition, the structural characteristics of telomerase-positive and ALT telomeres likely differ, providing another potential explanation for the differential roles for Ku in t-circle formation. A greater understanding of the precise mechanisms involved will require additional experimentation, however, the work by Li and coworkers provides striking evidence that Ku serves very specific roles at the telomere that can vary as the telomere biology varies, even in human cells, and suggests that in at least a subset of ALT cell lines, Ku is involved in the resolution of the telomere-induced genomic crisis that these cells have undergone during their clonal evolution.
